# Macrolide Resistance–associated 23S rRNA Mutation in *Mycoplasma genitalium*, Japan

**DOI:** 10.3201/eid1706.101055

**Published:** 2011-06

**Authors:** Yasushi Shimada, Takashi Deguchi, Keita Nakane, Mitsuru Yasuda, Shigeaki Yokoi, Shin-ichi Ito, Masahiro Nakano, Shin Ito, Hiroaki Ishiko

**Affiliations:** Author affiliations: Gifu University, Gifu, Japan (Y. Shimada, T. Deguchi, K. Nakane, M. Yasuda, S. Yokoi, S.-I. Ito, M. Nakano);; Mitsubishi Chemical Medience Corporation, Chiba, Japan (Y. Shimada, H. Ishiko);; iClinic, Sendai, Japan (S. Ito)

**Keywords:** bacteria, Mycoplasma genitalium, azithromycin, macrolide resistance, 23S rRNA, L4 ribosomal protein, L22 ribosomal protein, letter

**To the Editor**: *Mycoplasma genitalium* is now recognized as a serious pathogen in sexually transmitted infections (*1**,**2*). Azithromycin regimens have been commonly used for treatment of *M. genitalium* infections (*3*). However, failure of azithromycin treatment has been reported in cases of *M. genitalium*–positive nongonococcal urethritis (NGU) (*4**,**5*), and macrolide-resistant strains of *M. genitalium* have been isolated from case-patients in Australia, Sweden, and Norway for whom azithromycin treatment has failed (*4**,**5*). In these strains, mutations in the 23S rRNA gene were associated with macrolide resistance, and mutations in ribosomal protein genes L4 and L22 were also found (*5*). Surveillance for antimicrobial resistance of *M. genitalium* is essential to identify antimicrobial resistant strains and to then determine appropriate treatment. Coculture of patient specimens with Vero cells has improved the primary isolation rate of *M. genitalium* from clinical specimens and offered some current clinical strains for antimicrobial drug susceptibility testing (*6*). To determine their antimicrobial susceptibilities, a molecular real-time PCR method has been developed (*7**,**8*). However, isolating *M. genitalium* from clinical specimens and antimicrobial drug susceptibility testing of clinical isolates remain labor-intensive, time-consuming tasks. In addition, no methods are available to directly determine antimicrobial drug susceptibilities of *M. genitalium* in clinical specimens. To monitor macrolide susceptibilities in clinical strains of *M. genitalium* in Japan, therefore, we examined *M. genitalium* DNA found in the urine of men with NGU for the presence of macrolide resistance–associated mutations in the 23S rRNA gene and the ribosomal protein genes L4 and L22.

This retrospective study was approved by the Institutional Review Board of the Graduate School of Medicine, Gifu University, Gifu, Japan. We collected pretreatment urine specimens from 308 men with NGU who had visited a urologic clinic (iClinic) in Sendai, Japan, during 2006 through 2008 and stored the specimens at –70°C. Each man gave informed consent. Twenty-five of 58 urine specimens confirmed to be positive for *M. genitalium* by PCR-based assay were randomly chosen for this study and subjected to DNA purification. The 23S rRNA gene and the ribosomal proteins genes L4 and L22 of *M. genitalium* were amplified from the purified DNA by PCR as reported previously and then sequenced (*5*).

In 1 specimen, we found an A-to-G transition at nucleotide position 2072 in the 23S rRNA gene of *M. genitalium*, corresponding to position 2059 in *Escherichia coli* (Table). An A2059 (*E. coli* numbering) residue in region V of the 23S rRNA gene is critical for the binding of macrolides (*9*). Mutations of A2058, A2059, and other 23S rRNA residues within the macrolide-binding site can confer a high-level resistance to macrolides in several bacterial species, including *M. genitalium* (*5**,**9*). Therefore, *M. genitalium* strains that harbor the A2059G (*E. coli* numbering) mutation in the 23S rRNA gene could be highly macrolide resistant. We also found a T-to-G transition at nucleotide position 2199 in the 23S rRNA gene of *M. genitalium*, corresponding to position 2185 in *E. coli*, in 3 specimens, but this mutation has not been associated with macrolide resistance in other bacterial species (*9*).

**Table:** Mutations in the 23S rRNA gene and amino acid changes in L4 and L22 ribosomal proteins of 25 *Mycoplasma genitalium* strains in the pretreatment urine specimens of men with nongonococcal urethritis, Japan

No. urine specimens	Mutation in the 23S rRNA gene*	Amino acid change	
L4	L22
1	A2059G	–	Gly93Glu/Asp109Glu
1	T2185G	Val84Gly	–
1	T2185G	GLu128Gly	–
2	T2185G	–	–
1	–	Pro81Ser	–
1	–	Tyr135Pro	–
1	–	–	Ser81Thr
1	–	–	Met82Lys
1	–	–	Asn112Asp
1	–	–	Arg114Lys
14	–	–	–

*Nucleotide position in the 23S rRNA gene is according to *Escherichia coli* numbering. –, identical to the type strain.

We found amino acid changes in L4 and L22 ribosomal proteins in *M. genitalium* in 9 specimens. L4 and L22 ribosomal proteins each have extended loops, which converge to form a narrowing in the exit tunnel adjacent to the macrolide-binding site (*10*). Therefore, macrolide resistance–associated missense mutations in L4 and L22 tend to be localized to Gln62–Gly66 in L4 and Arg88-Ala93 in L22 of *E. coli*, which are closest to the macrolide-binding site (*10*). All of the amino acid changes in L4 of *M. genitalium* found in this study corresponded to those at the downstream regions from Gln62-Gly66 in L4 of *E. coli*. Of the amino acid changes in L22 of *M. genitalium*, the only Gly93Glu change found in *M. genitalium* harboring the A2059G (*E. coli* numbering) mutation in the 23S rRNA gene was located within the region corresponding to Arg88-Ala93 in L22 of *E. coli*. In this strain, therefore, the Gly93Glu change in L22 might contribute to the increase of macrolide resistance. The patient with NGU, whose specimen exhibited this strain of *M. genitalium* that harbored both the A2059G (*E. coli* numbering) mutation in the 23S rRNA gene and in which the Gly93Glu change in L22 was detected, was given a single dose of 1 g azithromycin and was clinically cured of NGU. However, the present study suggests that *M. genitalium* strains with high-level macrolide resistance might have already emerged in clinical settings in Japan. The emergence and spread of such a clinical mutant could threaten the ability of macrolides to treat *M. genitalium* infections. We should continue monitoring macrolide resistance of *M. genitalium* clinical strains. The nonculture approach used in our study will be useful until culturing of mycoplasmas from clinical specimens and antimicrobial drug susceptibility testing can be performed easily in laboratories.
